# Smoking Cessation on Periodontal and Peri-Implant Health Status: A Systematic Review

**DOI:** 10.3390/dj10090162

**Published:** 2022-08-31

**Authors:** Mario Caggiano, Roberta Gasparro, Francesco D’Ambrosio, Massimo Pisano, Maria Pia Di Palo, Maria Contaldo

**Affiliations:** 1 Department of Medicine, Surgery and Dentistry “Schola Medica Salernitana”, University of Salerno, Via S. Allende, 84081 Baronissi, Italy; 2 Department of Neuroscience, Reproductive Science and Dentistry, University of Naples Federico II, 80131 Naples, Italy; 3 Multidisciplinary Department of Medical-Surgical and Odontostomatological Specialities, University of Campania “Luigi Vanvitelli”, 80138 Naples, Italy

**Keywords:** smoking cessation, smoking cessations, tobacco, tobacco use, cigarette smoking, smokers, ex-smokers, non-smokers, periodontitis, periodontal disease, peri-implantitis, peri-implant disease

## Abstract

Since smoking is considered among the main risk factors for the onset and progression of periodontitis and peri-implantitis, the present systematic review aimed to evaluate the effect of smoking cessation on clinical, radiographic, and gingival crevicular periodontal parameters around natural teeth and dental implants in ex-smokers compared to current and non-smokers. The study protocol was developed based on the PRISMA guidelines, the research question was formulated according to the PICO model, and the literature search was conducted through PubMed/MEDLINE, Cochrane library, and BioMed Central databases. From the 916 title/abstracts initially identified, seven articles were included in the present systematic review and assessed for quality through the ROBINS-I tool. Reported findings on clinical and crevicular periodontal parameters around natural teeth were contrasting when comparing ex-smokers to current and non-smokers; thus, individualized recommendations for previous smoker periodontal patients are currently lacking. No data on radiographic parameters were retrieved. Similarly, data on periodontal parameters around dental implants were not available, highlighting the need for focused investigations assessing the role of both smoking habit and cessation on peri-implant health status and responsiveness to treatment.

## 1. Introduction

Periodontitis is a chronic inflammatory disease associated with the presence of microorganisms and sustained by host-mediated immune-inflammatory response [1,2,3,4], consequently establishing itself within periodontal tissues and causing their destruction with clinical attachment loss and bone loss [5,6,7,8] until tooth loss [9]. The inflammation of periodontal tissues, along with the dysbiotic phenomena of the periodontal microbiome [10,11,12,13], would also appear to play a role in the pathogenesis of systemic conditions and disorders of degenerative inflammatory and neoplastic nature [14,15,16,17,18,19], which, in turn, could influence the onset and, more importantly, the progression of periodontitis. The rate of progression of periodontitis is primarily estimated through both direct clinical and radiographic evidence of periodontal destruction assessed over time and indirect evidence related to biofilm accumulation. It is affected by the effect of glycemia and smoking, recognized as periodontitis grade modifiers [20]. In particular, smoking is also considered among the main risk factors for the onset of periodontitis [21,22]. Similarly, peri-implantitis, which is a microbially initiated destructive inflammation of the tissues surrounding dental implants which eventually determines bone loss until implant loss [23,24,25,26,27,28,29], shares common etiopathogenic pathways with periodontitis [30] and is also negatively affected by habitual smoking [31,32].

However, the biological and molecular mechanisms underlying the negative association between smoking and the health status of periodontal and peri-implant tissues has not yet been defined; thus, at the current state of knowledge, there are no preventive or therapeutic approaches in periodontal practice individualized for periodontal subjects who smoke. Coherently, smoking cessation may be regarded as the best feasible intervention for reducing the risk of onset and progression of both periodontitis and peri-implantitis [31]. Although multiple pharmacological, non-pharmacological, and combined approaches have been developed for smoking cessation to achieve short-term smoking abstinence and relapse avoidance, as a part of inter-professional primary and secondary prevention strategies [4,33,34,35], the impact of smoking cessation on periodontal and peri-implant health status has been rarely investigated.

Therefore, the present systematic review aimed to assess the effect of smoking cessation on clinical, radiographic, and gingival crevicular periodontal parameters around natural teeth and dental implants in ex-smokers compared to non-smokers and current smokers.

## 2. Materials and Methods

### 2.1. Study Protocol

The present study was conducted under the Preferred Reporting Items for Systematic Reviews and Meta-analyses (PRISMA) statement [36,37], available at http://www.prisma-statement.org/ (accessed on 15 July 2022).

Question formulation, search strategy, and study selection definition were performed according to the PICO model [38] (https://linkeddata.cochrane.org/pico-ontology (accessed on 15 July 2022)). The question of the present systematic review was “Does smoking cessation positively affect periodontal and peri-implant health status?” focusing on:

P—Population: ex-smokers (traditional “TS” and Heat-Not-Burn “HNB” tobacco and electronic nicotine delivery systems “E-cigs” smokers);

I—Intervention: smoking cessation;

C—Comparison: smoking habit/no previous history of tobacco smoke (current smokers “CS”/non-smokers “NS”);

O—Outcome: clinical, radiographic, and inflammatory periodontal parameters around natural teeth and implants.

### 2.2. Search Strategy

Articles published and in press in the English language were electronically searched by two independent reviewers (F.D.A., M.P.D.P.) until 15 July 2022, with no restrictions concerning dates of coverage and publication status, across MEDLINE/PubMed, Cochrane library, and BioMed Central databases by applying the following keywords:

1. Periodontal disease OR periodontitis OR peri-implant disease OR peri-implantitis OR tooth loss OR dental implant OR implant loss OR clinical attachment loss OR probing depth OR plaque index OR gingival index OR bleeding on probing OR marginal bone loss OR TNF-a OR IL-1b OR IL-4 OR IL-6 OR IL-8 OR IL-9 OR IL-10 OR IL-13 OR IFN-y OR MMP-1 OR MMP-8 OR RANKL OR OPG).

AND:

2. Smoking cessation OR stop smoking OR quit smoking OR cessation of smoking OR stopping smoking OR quitting smoking OR ex-smokers OR exsmokers).

Reference lists of eligible articles were also screened, and a further additional literature search was conducted.

### 2.3. Study Selection

Two reviewers (F.D.A., M.P.D.P) independently performed study selection, involving a third reviewer (M.C.) in case of discrepancies.

Title and abstract selection was performed for all papers identified through the electronic literature search and potentially relevant full-texts were also retrieved by contacting the authors.

Full texts were screened for potentially eligible and ambiguous abstracts according to the inclusion/exclusion criteria shown in Table 1.

### 2.4. Data Collection and Synthesis

Three independent reviewers (F.D.A., M.P.D.P, M.C.) extracted data twice and collected them in a dedicated form based on those proposed for RCT and non-RCT intervention reviews [39] (https://dplp.cochrane.org/data-extraction-forms (accessed on 15 July 2022)). No further processes were performed to obtain or confirm data from the investigators.

Data collected for the studies included in the present systematic review concerned study source (first author, year of publication and journal, funding) and design, participants (number, gender, and age; comorbidities, smoking habit duration), intervention (smoking cessation duration), comparison (current smokers/non-smokers), and periodontal outcomes around natural teeth and implants (clinical and radiographic indices, gingival crevicular inflammatory mediator levels, and conclusions).

Descriptive statistical analyses were conducted using Microsoft Excel software 2019 (Microsoft Corporation, Redmond, WA, USA).

### 2.5. Quality Assessment

Study quality assessment was performed through the ROBINS-I (“Risk Of Bias In Non-randomized Studies of Interventions”) tool, based on biases due to confounding, participant selection, classification of interventions, deviations from intended interventions, missing data, outcome measurements, and selection of the reported result [40].

## 3. Results

### 3.1. Study Selection

In total, 1086 titles/abstracts were initially retrieved through the electronic search, specifically 897 from MEDLINE/PubMed, 3 from BioMed Central databases, and 186 from the Cochrane library, respectively. Duplicates were eliminated and 916 potentially pertinent title/abstracts were identified, of which24 records concerned periodontal health conditions around natural teeth and around dental implants.

**Figure 1:**
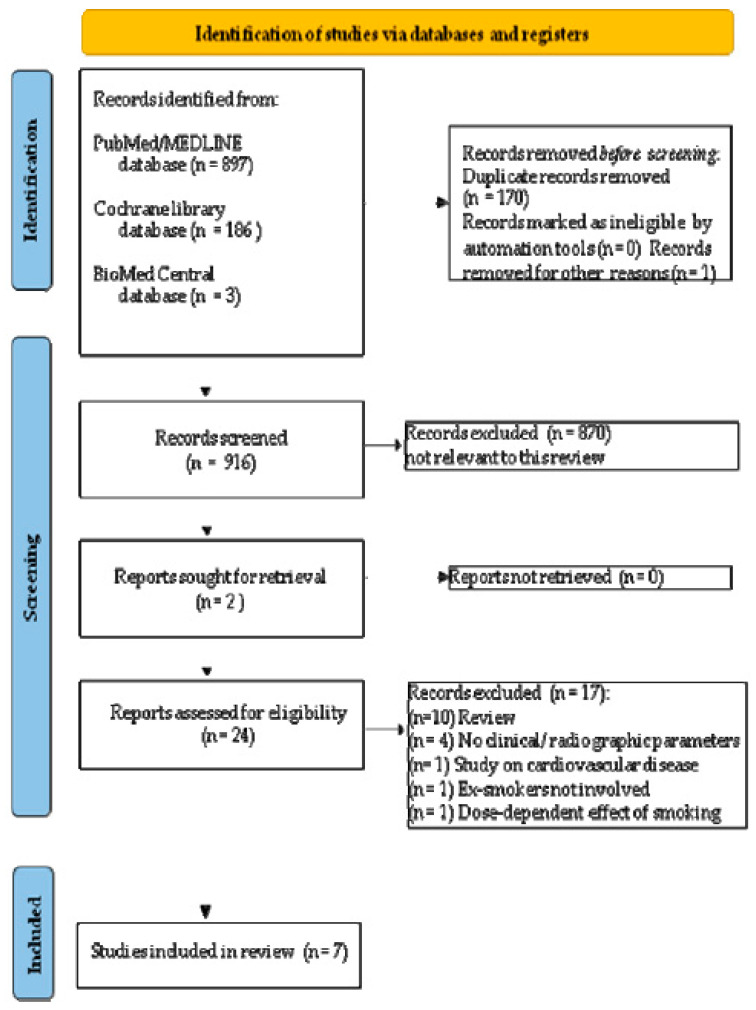
PRISMA 2020 flow diagram for new systematic reviews which included searches of databases and registers only.

After title/abstract screening, 17 records were excluded because they were not pertinent, including 10 reviews [41,42,43,44,45,46,47,48,49,50], 4 studies without clinical data, and 3 studies that did not meet inclusion criteria [51,52,53,54,55,56,57], as shown in Figure 1 (and synthesized in the table of the studies excluded with reasons for exclusion available as Supplementary Materials). Subsequently, seven full-texts were screened which did not require contacting the authors. Based on eligibility criteria, seven studies [58,59,60,61,62,63,64] were finally included in the present systematic review, as illustrated in Figure 1.

### 3.2. Study Characteristics and Descriptive Data Analysis

Descriptive analysis of the studies included in the present systematic review is detailed in Table 2.

The extracted data, synthesized in Table 3, all concerned periodontal parameters around natural teeth and none of the data concerned periodontal parameters around dental implants. Radiographic periodontal parameters were not described in the records retrieved from the literature. Due to the heterogeneity of the included studies and the lack of randomized controlled trials, it was not possible to conduct a metanalysis.

### 3.3. Quality Assessment

The studies currently considered were assessed for quality [40], as shown in Table 4. In detail, Jeong et al., 2020 [59], used a different measurement parameter for periodontal disease, the CPI, that might be considered a bias for measurement of outcomes.

## 4. Discussion

It has long been known that smoking habit increases the risk of periodontitis onset [31], by up to 85% as per Leite et al. [41], and progression [9]. In addition, smoking has been commonly regarded as potentially detrimental for dental implant survival and success [5,8,23,65] and is considered as a negative predictor for tooth and dental implant loss [32,65]. Therefore, the present systematic review aimed to evaluate the effect of smoking cessation on clinical, radiographic, and gingival crevicular periodontal parameters around natural teeth and dental implants in ex-smokers compared to non-smokers and current smokers.

Even so, the seven studies included in the present systematic review only evaluated the periodontal state around natural teeth after periodontal treatment, while none of the studies included the state around implants. This finding suggests the need for extensive additional research, especially considering that very little evidence is currently available on the role of smoking habit on peri-implant tissue health [24,65,66]. Indeed, conflicting results have been reported in the literature. Specifically, Koldsland et al. [67] and Roos-Jansåker et al. [68,69] failed to detect a significant association between smoking and peri-implant disease prevalence and implant loss, respectively.

Data on former and current conventional tobacco smokers were collected and analyzed; those concerning electronic devices were included but qualitatively analyzed separately. Conversely, no data concerning Heat-Not-Burn tobacco product smokers have been retrieved, though increasingly common [32], probably due to their recent use. In addition, findings from the so-called “oscillators”, i.e., previous smokers with relapse, were excluded from the investigated population to reduce the risk of bias due to the selection of participants.

In detail, periodontal tissue destruction around natural teeth was known to be greater in current smokers [70] compared to non-smokers and ex-smokers, as also confirmed by Costa et al. [60,61], even though Karaaslan et al. [58] found similar CAL values and a similar percentage of sites with CAL values ≥ 5 mm among smokers, non-smokers, and ex-smokers. As a counterpart, tooth loss as a consequence of periodontitis had a higher rate of occurrence in current and ex-smokers [60,61] compared to non-smokers, especially in younger males [62]. However, Costa et al. recently found that the amount of teeth loss due to periodontitis was higher in ex-smokers compared to current smokers and non-smokers; this observation may be likely ascribable to the higher mean age of the ex-smokers [61]. Notably, a negative dose-dependent association between smoking habit and the number of residual natural teeth, declining after approximately 20 years from smoking cessation, was described by Dietrich et al. [62]. This timeframe may, if validated, aid in estimating periodontitis onset odds ratio decline in ex-smokers, and accordingly guide maintenance recall interval planning. Radiographic periodontal parameters assessing alveolar bone loss were described neither around natural teeth nor dental implants in ex-smokers, so no comparison could be made with radiographic findings registered as better or worse in smokers compared to non-smokers [16,30,32]. However, considering that CAL and bone loss quantify historical tissue destruction [4,9,21,22], significant differences should not be expected between smokers and subjects quitting smoking recently, while an improvement in PD values may be anticipated.

Accordingly, Costa et al. [60] and Beklen et al. [64] reported lower periodontal probing depth values and a lower percentage of sites with a PD ≥ 5 mm in ex-smokers and non-smokers compared to current smokers. No differences in PD values were in anyway observed by Karaaslan et al. and Liu et al. [58,63] in relation to a smoking habit or smoking cessation. Such contrasting results should also be evaluated in conjunction with individual periodontal inflammatory indices, potentially affecting gingival hypertrophy, and with local causative factors such as biofilm also supporting superficial tissue edema.

In detail, a significantly lower mean number of sites with BOP+ was described in smokers vs. ex-smokers and non-smokers [60,61], likely due to the vasoconstriction observable within gingiva and, conceivably, peri-implant mucosa, secondary to both traditional and Heat-Not-Burn tobacco products [32,62,71]. Furthermore, GI values were reported to be higher in ex-smokers compared to current smokers [58,63,64].

Beklen et al. [60] and Costa et al. [64] described higher PI values in smokers compared to ex-smokers and non-smokers. This finding, combined with the hypothesis that nicotine may favor the proliferation of *A. actinomycetemcomitans* and *P. gingivalis* which are suspected periodontal pathogens species, may support the wider periodontal destruction observed in smokers. Similarly, a greater microbial load, particularly composed of periodontal pathogens, has been detected in peri-implant sulci in smokers [28,65]. Given that peri-implant biofilm shares microbial species with periodontal ones, both in physiological and pathological conditions, it has been accordingly proposed that residual teeth and periodontal tissues may act as potential reservoirs of pathogens which eventually colonize peri-implant tissues in subjects with active periodontitis, especially if smokers [28,65,72]. However, in contrast, Karaaslan et al. [58] and Liu et al. [63] did not find significant differences in plaque amounts between current, former, and non-smokers; therefore, no definitive conclusions could be drawn.

It is worth noting that even if the smoking habit has also been described to negatively affect periodontal therapy outcomes in smokers [73], considering clinical, radiographic, and crevicular parameters around both natural teeth and dental implants [32], no relevant data were currently extracted concerning ex-smokers undergoing periodontal treatment.

Both cumulative smoking exposure and duration of smoking cessation were only reported to be significantly associated with periodontitis by Costa et al. [60,61]. However, a paucity of evidence exists to describe the role of the number of cigarettes/day and the time interval since smoking cessation on the potential improvement of periodontal and peri-implant parameters. Similarly, the estimate of the reduction is still debated regarding former vs. current and non-smokers and the risk of periodontitis and peri-implantitis onset and worsening; such an estimate has been computed, instead, for smoking cessation in relation to life expectancy, which increases by 10, 9, and 6 years for those who quit smoking at 30, 40, and 50 years [74], respectively. In this regard, Alharti et al., 2018 [57], reported a higher prevalence of periodontitis of up to 35% in smokers, compared to 19% in previous smokers and 13% in non-smokers, and estimated a reduction in the risk of periodontitis progression of 3.9% for each year of smoking cessation.

The results of the present study, that turned out to be so contrasting, could be explained by the long-standing debate on the pathogenic role of smoking in the onset and progression of periodontitis and peri-implantitis. Indeed, the causative role of smoking in the genesis of these diseases, which are microbially associated inflammations, has long been supported by evidence revealing an intrinsic chemical and mechanical capacity of smoking to compromise periodontal health status. In contrast, multiple pieces of evidence suggested that it was instead the poor oral care of smokers, and thus the accumulation of biofilm, that determined the onset and progression of periodontitis. At the current state of knowledge, smoking has been found capable of reducing host defenses and indirectly facilitating the action of virulence factors of suspected pathogenic species within the biofilm. Smoking has been shown to induce the production of proinflammatory cytokines and enzymes with a destructive effect within periodontal tissues in addition to facilitating, as mentioned above, colonization by pathogenic species [75,76,77]. In detail, crevicular proinflammatory biomarkers have been presently found and recorded by several authors since they may be useful tools for diagnostic and prognostic purposes in both periodontal and peri-implant disease. In particular, proinflammatory cytokines such as Tumor Necrosis Factor-alpha and Interleukin-1b are secreted by macrophages in response to lipopolysaccharide contained in bacterial membranes. Consequently, macrophages can activate osteoclastogenesis, causing bone resorption, and induce fibroblast apoptosis, contributing to clinical attachment loss. Crevicular IL-1b and TNF-a levels detected in current smokers were found to be significantly higher, particularly in traditional tobacco smokers vs. E-cigs smokers [32], compared to former and non-smokers.

The main limitations of the present systematic review rely upon the few relevant articles identified from the literature search, including the evaluation of periodontal outcomes in ex-smokers. In addition, no data could be retrieved regarding clinical periodontal parameters around dental implants or radiographic parameters around the dental elements and around the implants. The data extracted and analyzed were severely lacking and contrasting and were derived from studies that included smokers with different smoking habits and smoking cessation durations, precluding comparison among the groups. In most of the studies, the duration of previous smoking habits and the type of smoking, as well as the duration of smoking cessation, were often not specified. Moreover, data concerning periodontal outcomes in ex-smokers of Heat-Not-Burn tobacco products were not found, probably due to the recent introduction of these tobacco systems. Therefore, data were thus found to be very deficient, and it was not possible to perform the meta-analysis.

Nevertheless, the presented results clearly highlight the need for further investigations assessing the potentially beneficial role of smoking cessation on periodontal conditions around teeth and dental implants. In more detail, future research may identify a time cut-off for detecting such improvements, estimate their magnitude in relation to the time interval from smoking cessation, and thus favor personalized planning of initial and maintenance periodontal treatments in ex-smokers. Moreover, future investigations may also point out the odds ratio of periodontitis and peri-implantitis onset in ex-smokers compared to current smokers and non-smoking periodontally healthy subjects, thus improving periodontitis and peri-implantitis prevention. A similar estimate might also be computed for the progression of such diseases in periodontal subjects. Personalized prevention strategies may be even more relevant in those subjects considered at higher risk of periodontitis and peri-implantitis onset and progression, such as those suffering from comorbidities [4,10,14,15,30,33,78,79,80], especially diabetes [17].

## 5. Conclusions

A paucity of evidence describes the effect of smoking cessation on clinical, radiographic, and crevicular periodontal parameters around natural teeth. Even fewer data describe the effect of smoking cessation on periodontal treatment outcomes; therefore, individualized recommendations for periodontal patients who are smokers or ex-smokers, with or without comorbidities, are currently lacking. Thus, further investigation should point out the role of smoking cessation on periodontally healthy subjects, as well as on those suffering from periodontitis, aiding in periodontal treatment planning in active and, above all, maintenance phases.

No data were retrieved concerning periodontal parameters around dental implants, highlighting the need for focused investigations assessing the role of both smoking habit and cessation on peri-implant health status and responsiveness to treatment.

## Figures and Tables

**Table 1:** Inclusion and exclusion criteria related to source, characteristics, population, intervention, comparison, and outcome(s) of relevant studies.

Study	Inclusion Criteria	Exclusion Criteria
**Characteristics**		
**Type**	Clinical	Narrative reviews
		Books and chapters
		Conference papers
		Oral communications
		In vitro
		Pre-clinical in vivo
**Design**	Cross-sectional	Case series
	Case-control	Case reports
	Retrospective	
	Prospective	
	Randomized Clinical Trials (RCT)	
**Sample size**	≥50	<50
**Population**	Periodontal subjects	Periodontally healthy subjects
**Age**	≥18 years old	<18 years old
**Gender**	No restrictions	No restrictions
**Characteristics**	Ex-smokers	Smokers
		Non-smokers
		Pregnancy; lactation
**Comorbidities**	Any	Neoplastic disease
		Medication-related osteonecrosis of the jaws
		Systemic disease affecting bone metabolism or periodontal/peri-implant disease
		Non-surgical/surgical periodontal treatment (within <3 months)
		Radiations (head and neck)
		Antibiotics, corticosteroids (within 3 months)
		Drugs affecting bone metabolism
**Treatments**	Non-surgical/surgical periodontal treatment (within ≥3 months)	
**Intervention**	Smoking cessation (ex-smokers)	No discontinuing tobacco smoking
**Comparison**	No smoking cessation (current smokers)	
	No smoking habit (non-smokers)	
**Outcome(s)**	Periodontal and peri-implant status	Endodontic-periodontal lesions
		Endodontic lesions
		Failure of osseointegration
	Clinical Attachment Level (CAL)	
**Clinical periodontal parameters**	Periodontal Probing Depth (PPD)	
	Plaque Index (PI)	
	Gingival Index (GI)	
	Bleeding on Probing (BOP)	
	Tooth loss; implant loss	
**Radiographic periodontal parameters**		
	Radiographic bone loss around natural teeth (RBL)	
	Marginal bone loss around dental implants (MBL)	
**Other periodontal parameters**	Gingival crevicular (GC) inflammatory mediators	

**Table 2:** Data extracted and collected from the studies included in the present systematic review: general information; methods; periodontal outcomes; conclusion(s).

Included Studies	Methods	Periodontal Parameters Around Natural Teeth Statistically Significant (*p* < 0.05)	Conclusion(s)
Author Year [Reference] Journal Study design	**Population**	**Clinical**	
	Participants (n.)	Clinical Attachment Level (CAL)	
	Age (y.o.)		
	Male/female (M/F)	Periodontal Probing Depth (PPD)	
	Periodontal status		
	Comorbidities	Bleeding on Probing (BOP)	
	Smoking habit duration (years)		
	Smoking habit characteristics: traditional tobacco;	Gingival Index (GI)	
	Heat-Not-Burn tobacco; electronic nicotine delivery systems (E-cigs);	Plaque Index (PI)	
	Cigarettes or equivalent n./day		
	**Intervention**	Tooth loss	
	Smoking cessation (duration)	Implant loss	
	(ex-smokers)		
	**Comparison**	**Radiographic**	
	Ongoing smoking habit (smokers)	Radiographic bone loss (RGB)	
	No smoking habit (non-smokers)		
	**Procedure(s)**	**Gingival crevicular (GC) inflammatory mediators**	
	Non-surgical periodontal treatment	Any	
	Surgical periodontal treatment		
Karaslaan et al., 2020 [58] Aust. Dent. J. Case-control study	Study participants (n.57)	**Clinical**	GI was significantly higher among E-cigs smokers and ex-smokers compared to tobacco smokers GI was significantly lower among E-cigs smokers compared to ex-smokers
	Age (35.19 +/− 2.23)	CAL	
	Male/female (39/18)	GI	
	Periodontal subjects	PI	
	Comorbidities: MD		
		**Radiographic**	
	Smoking habit duration: MD	MD	
	Smoking habit characteristics: tobacco smokers (13.95 +/−3.01 years)		
	E-cigs smokers (2.32 +/− 0.75 years)	**GC Inflammatory mediators**	
		Interleukin-8	
	**Intervention**	Tumor Necrosis Factor-alfa	
	Smoking cessation (duration MD)	Glutathione peroxidase	
		8-hydroxydeoxyguanosine	
	**Comparison**		
	- tobacco smokers with periodontitis		
	- E-cigs smokers with periodontitis		
	- ex-smokers with periodontitis		
	**Procedure(s)**		
	Non-surgical periodontal treatment		
Jeong et al., 2020 [59] J. Periodont. Case-control study	Study participants (n.13551)	**Clinical**	Periodontitis rate was significantly higher in tobacco smokers and E-cigs smokers compared to ex-smokers and smokers
	Age (divided into range of 10 years)	CPI divided into 0–4 points	
	Male/female (5715/7836)	0 for healthy periodontal tissue	
	Periodontal status: MD	1 (bleeding periodontal tissue)	
	Comorbidities: MD	2 (gingival biofilm)	
		3 (3.5 ≤ pocket depth < 5.5 mm)	
	Smoking habit duration: MD	4 (pocket depth ≥ 5.5 mm).	
	Smoking habit characteristics: Tobacco smokers	A score of 3–4 denotes periodontal disease	
	E-cigs smokers		
		**Radiographic**	
	**Intervention**	MD	
	Evaluation of periodontal diseases among the group		
		**GC Inflammatory mediators**	
	**Comparison**	MD	
	- non-smokers		
	- tobacco smokers		
	- E-cigs		
	- ex-smokers		
	**Procedure(s)**		
	Non-surgical periodontal treatment		
Costa et al., 2013 [60] J. Oral Sci. Cross-sectional study	Study participants (n.705)	**Clinical**	Cumulative smoking exposure and duration of smoking cessation were significantly associated with periodontitis
	Age (35–65 y.o.)	CAL	
	Male/female (341/364)	PI	
	Periodontal status: MD	BOP	
	Comorbidities: MD	PPD	
	Smoking habit duration:	**Radiographic**	
	in ex-smokers (28.6 +/− 12.7 years) and in smokers (35.5 + 7 − 14.8 years)	MD	
	Smoking habit characteristics: non-smokers		
	tobacco smokers	**GC inflammatory mediators**	
	ex-smokers	MD	
	**Intervention**		
	Smoking cessation (duration MD)		
	**Comparison**		
	- non-smokers		
	- smokers		
	- ex-smokers		
Costa et al. 2019 [61] J. Periodont. Cohort study	Study participants (n.142)	**Clinical**	Smoking negatively affects periodontitis and, in particular, smoking cessation positively affects periodontitis
	Age (MD)	CAL	
	Male/female (MD)	BOP	
	Periodontal status: MD	PPD	
	Comorbidities: diabetes	PI	
	Smoking habit duration: MD	**Radiographic**	
	Smoking habit characteristics: non-smokers	MD	
	tobacco smokers		
	ex-smokers	**GC inflammatory mediators**	
		MD	
	**Intervention**		
	Smoking cessation (6 years)		
	**Comparison**		
	- smokers		
	- non-smokers		
	- ex-smokers		
Dietrich et al. 2015 [62] J. Dent. Res. Prospective cohort study	Study participants (n.23376)	**Clinical**	Smoking habit had a stronger association with tooth loss in M > F and in younger > older subjects Smoking cessation was associated with a reduction in tooth loss risk in approximately 20 years
	Age (non-smokers, 50.3 ± 8.8; ex-smokers, 50.5 ± 9.0; smokers, 47.2 ± 8.7)	Tooth loss	
	Male/female (9032/14,344)		
	Periodontal status: MD	**Radiographic**	
	Comorbidities: diabetes and hypertension	MD	
	Smoking habit duration: MD	**GC Inflammatory mediators**	
	Smoking habit characteristics: non-smokers	MD	
	tobacco smokers		
	ex-smokers		
	**Intervention**		
	Smoking cessation (duration MD)		
	**Comparison**		
	- non-smokers		
	- tobacco smokers		
	- ex-smokers		
Liu et al., 2015 [63] J. Perio. Res. Prospective study	Study participants (n.122)	**Clinical**	No significant differences in GC Matrix Metalloproteasis-8 and Matrix Metalloproteasis-9 were detected between smokers, ex-smokers (for 1 year), and non-smokers This 1-year prospective smoking cessation study shows GC Interleukin-1b could have a positive relationship with nicotine and cotinine levels in saliva
	Age (MD)	GI	
	Male/female (122/0)	PI	
	Periodontal status: MD		
	Comorbidities: MD	**Radiographic**	
		MD	
	Smoking habit duration: MD		
	Smoking habit characteristics: n.13 non-smokers	**GC inflammatory mediators**	
	n.11 ex-smokers	Matrix Metalloproteasis-8	
	n.9 smokers	Matrix Metalloproteasis-9	
	n.6 oscillators	Interleukin-1b	
		Cotinine	
	**Intervention**	Nicotine	
	Smoking cessation (1 year)		
	**Comparison**		
	- non-smokers (n.13)		
	- ex-smokers (n.11)		
	- smokers (n.9)		
	- oscillators (n.6)		
Beklen et al., 2021 [64] TobInduc. Dis. Cross-sectional study	Study participants (n.226)	**Clinical**	Higher PI was found in smokers (2.78 ± 0.92) compared to non-smokers (1.0 ± 0.6) and ex-smokers (1.1 ± 0.8) PD values were significantly (*p* < 0.05) higher in smokers (5.6 ± 1.9) compared to non-smokers (1.6 ± 0.8) and ex-smokers (2.4 ± 1.3)
	Age (>18 years)	PI	
	Male/female (90/136)	GI	
	Periodontal status: MD	PD	
	Comorbidities: MD		
		**Radiographic**	
	Smoking habit duration: MD	MD	
	Smoking habit characteristics: non-smokers		
	tobacco smokers	**GC Inflammatory mediators**	
	ex-smokers	MD	
	**Intervention**		
	Smoking cessation (duration MD)		
	**Comparison**		
	- non-smokers		
	- tobacco smokers		
	- ex-smokers		

Abbreviations: current smokers, CS; traditional tobacco smokers, TS; electronic cigarette or electronic cigarette smokers, E-cigs; non-smokers, NS; years old, y.o.; missing data, MD; Probing Depth, PD; clinical attachment loss, CAL; Plaque Index, PI; Bleeding on Probing, BOP; Gingival Index, GI; Community Periodontal Index, CPI; gingival crevicular, GC.

**Table 3:** Synthesis of the periodontal parameters around natural teeth reported in the studies included in the present systematic review.

Periodontal Parameter	Main Result(s)	Author, Year
CAL	Mean CAL values and the percentage of sites with a CAL value ≥ 5 mm and tooth loss were lower in ex-smokers and non-smokers compared to smokers;	Costa et al., 2013 [60]
	Mean CAL values and the percentage of sites with a CAL value ≥ 5 mm and tooth loss were lower in ex-smokers and non-smokers compared to smokers;	Costa et al., 2019 [61]
	Mean CAL values were not significantly different among ex-smokers, smokers, and non-smokers.	Karaaslan et al., 2020 [58]
PI	Mean PI values were lower in ex-smokers and non-smokers compared to smokers;	Costa et al., 2013 [60]
	Mean PI values were not significantly different among ex-smokers, smokers, and non-smokers;	Liu et al., 2015 [63]
	Mean PI values were not significantly different among ex-smokers, smokers, and non-smokers;	Karaaslan et al., 2020 [58]
	Mean PI values were lower in ex-smokers (1.1 ± 0.8) and non-smokers (1.0 ± 0.6) compared to smokers (2.78 ± 0.92);	Beklen et al., 2021 [64]
	Mean PI values were lower in ex-smokers and non-smokers compared to smokers (PI values for smokers > ex-smokers > non-smokers).	Costa et al., 2019 [61]
BOP	The mean number of sites with BOP was significantly lower in smokers compared to ex-smokers and non-smokers;	Costa et al., 2013 [60]
	The mean number of sites with BOP was significantly lower in smokers compared to ex-smokers and non-smokers.	Costa et al., 2019 [61]
PD	Mean PD values were not significantly different among ex-smokers, smokers, and non-smokers;	Karaaslan et al., 2020 [58]
	Mean PD values and he percentage of sites with a PD ≥ 5 mm were lower in ex-smokers and non-smokers compared to smokers;	Costa et al., 2013 [60]
	Mean PD values and the percentage of sites with a PD ≥ 5 mm were lower in ex-smokers (2.4 ± 1.3) and non-smokers (1.6 ± 0.8) compared to smokers (5.6 ± 1.9);	Beklen et al., 2021 [64]
	Mean PD values were not significantly different among ex-smokers, smokers, and non-smokers.	Liu et al., 2015 [63]
GI	Mean GI values were not significantly different among ex-smokers, smokers, and non-smokers at baseline;	Liu et al., 2015 [63]
	GI values were lower in ex-smokers (1.9 ± 1.0) and non-smokers (0.5 ± 0.4) compared to smokers (2.5 ± 0.5);	Beklen et al., 2021 [64]
	GI values were significantly higher in ex-smokers and electronic cigarette smokers compared to smokers and were significantly lower in electronic cigarette smokers compared to ex-smokers.	Karaaslan et al., 2020 [58]
Tooth loss	Tooth loss was lower in ex-smokers and non-smokers compared to smokers;	Costa et al., 2019 [61]
	Tooth loss was significantly higher in ex-smokers and smokers compared to non-smokers;	Costa et al., 2013 [60]
	A negative dose-dependent association between cigarette smoking, smoking cessation, and number of natural teeth was found at baseline.	Dietrich et al., 2015 [62]
Tumor Necrosis Factor-alfa (TNF-a)	GC TNF-a levels were significantly higher in smokers (4.20 +/− 0.14) compared to electronic cigarette smokers; no data concerning ex-smokers.	Karaaslan et al., 2020 [58]
Interleukin-1b (IL-1b)	GC IL-1b was significantly lower (*p* = 0.007) in ex-smokers, non-smokers, and oscillators compared to smokers at 2-month follow-up.	Liu et al., 2015 [63]
Interleukin-8 (IL-8)	GC IL-8 levels were significantly higher in ex-smokers (70.47 +/− 2.76) and electronic cigarette smokers compared to smokers.	Karaaslan et al., 2020 [58]
CPI	Periodontal disease was more prevalent in electronic cigarette smokers and tobacco smokers compared to non-smokers; no data concerning ex-smokers.	Jeong et al., 2020 [59]
Matrix metalloproteasis-8 and -9 (MMP-8 -9)	GC MMP-8 and MMP-9 were not significantly different between smokers and ex-smokers (for 1 year).	Liu et al., 2015 [63]
Glutathione peroxidase	GC glutathione peroxidase levels were significantly higher in ex-smokers compared to tobacco smokers, electronic cigarette smokers, and ex-smokers.	Karaaslan et al., 2020 [58]

Abbreviations: traditional tobacco smokers, TS; electronic cigarette or electronic cigarette smokers, E-cigs; non-smokers, NS; years old, y.o.; missing data, MD; Probing Depth, PD; clinical attachment loss, CAL; Plaque Index, PI; Bleeding on Probing, BOP; Gingival Index, GI; Community Periodontal Index, CPI; gingival crevicular, GC.

**Table 4:** Risk of bias for the studies included in the systematic review: ‘Yes’ indicating a low risk of bias, ‘Probably yes’ indicating a moderate risk of bias, ‘Probably no’ indicating a serious risk of bias, ‘No’ indicating a critical risk of bias, and ‘No information’ indicating that no information was available.

Studies	Bias Due to Confounding	Bias in Selection of Participants	Bias in Measurement Classification of Interventions	Bias Due to Deviations from Intended Interventions	Bias Due to Missing Data	Bias in Measurement of Outcomes	Bias Due to Selection of the Reported Result
Costa et al., 2013 [60]	Yes	Yes	Yes	Yes	Yes	Yes	Yes
Costa et al., 2019 [61]	Probably yes	Yes	Yes	Yes	Yes	Yes	Yes
Karaaslan et al., 2020 [58]	Yes	Yes	Yes	Yes	Yes	Yes	Yes
Liu et al., 2015 [63]	Probably no	Yes	Yes	Yes	Yes	Yes	Yes
Dietrich et al., 2015 [62]	Yes	Yes	Yes	Yes	Yes	Yes	Yes
Beklen et al., 2021 [64]	Yes	Yes	Yes	Yes	Yes	Yes	Yes
Jeong et al., 2020 [59]	Probably no	Yes	Yes	Yes	Yes	Probably yes	Yes
Risk of bias judgements	Serious	Low	Low	Low	Low	Moderate	Low

## Data Availability

Reported data are available on MEDLINE/PubMed, Scopus, Cochrane library, and BioMed Central databases.

## Supplementary Materials

The following supporting information can be downloaded at: https://www.mdpi.com/article/10.3390/dj10090162/s1, Table S1: Studies excluded and reasons for exclusion. Reference [41,42,43,44,45,46,47,48,49,50,51,52,53,54,55,56,57] is cited in the supplementary materials.
