# Supplementary material for: Smoking Cessation on Periodontal and Peri-Implant Health Status: A Systematic Review
Source: Dent J (Basel). 2022 Aug 31;10(9):162. doi: 10.3390/dj10090162 (PMC9497918; doi:10.3390/dj10090162)
Supplement: Supplementary file 1 [file dentistry-10-00162-s001.zip › dentistry-1876947-supplementary.pdf]

Table S1. Studies excluded and reasons for exclusion.

Authors, year	Reason for exclusion
Leite et al., 2019 [41]	Review
Souto et al., 2019 [42]	Review
Ramseier et al., 2020 [43]	Review
Gugnani et al., 2020 [44]	Review
Al Ansari et al., 2020 [45]	Review
Chambrone et al., 2010 [46]	Review
Chambrone et al., 2013 [47]	Review
Fiorini et al., 2014 [48]	Review
Holliday et al ., 2021 [49]	Review
Alexandridi et al., 2018 [50]	Review
Preshaw et al., 2005 [51]	Studies without clinical/ radiographic parameters
Shimazaki et al., 2006 [52]	Prospective cohort study on cardiovascular disease
Peruzzo et al, 2016 [53]	No ex-smokers included
Nemmar et al., 2021 [54]	Studies without clinical/ radiographic parameters
More et al., 2021 [55]	Studies without clinical/ radiographic parameters
Ravidà et al., 2020 [56]	Dose-dependent effect of smoking
ALHarthi et al., 2018 [57]	Studies without clinical/ radiographic parameters
